# EYE-ECG2: Addressing medical student feedback in an RCT with eye-tracking videos featuring cued retrospective reporting and modified learning sequences for ECG interpretation skills training

**DOI:** 10.3205/zma001771

**Published:** 2025-09-15

**Authors:** Aline D. Scherff, Stefan Kääb, Martin R. Fischer, Markus Berndt

**Affiliations:** 1 LMU University Hospital, LMU Munich, Institute of Medical Education, Munich, Germany; 2 LMU University Hospital, LMU Munich, Department of Medicine I, Munich, Germany

**Keywords:** eye-tracking, learning, electrocardiography, medical students, clinical reasoning, diagnosis

## Abstract

**Objective:**

This study aimed to evaluate the effectiveness of an enhanced eye-tracking video intervention (EYE-ECG2) in improving ECG interpretation skills of medical students. Building on the foundational EYE-ECG1 study [Scherff et al. GMS J Med Educ. 2024], which tested the utility of expert eye movement modelling and retrospective think-aloud commentary for ECG skill acquisition, this follow-up study introduces modifications designed to optimise learning outcomes.

**Methods:**

A randomised controlled trial was conducted with medical students (*N*=94) allocated to either the control group (TAU) with standard ECG interpretation training, consisting of a validated set of 4 ECG cases for ECG interpretation; or the intervention group (INT) who additionally received the EYE-ECG2 video. The EYE-ECG2 video included refined ECG gaze cues and Cued Retrospective Reporting (CRR) by a senior cardiology expert. Performance was assessed pre- and post-training. Data were analysed to compare improvements in diagnostic accuracy, decision-making processes and student feedback were evaluated.

**Results:**

The results of the previous study were successfully replicated, showing an overall significant learning benefit from the cases and a moderate, yet nonsignificant tendency for INT>TAU (ΔM=0.80-2.42%; *p*=.79-.30). Randomised case presentation attenuated the special role of learning case 1 observed previously in EYE-ECG1. Exploration of student feedback showed a largely positive or neutral evaluation (74%), and a prior cardiological clerkship was a distinguishing factor resulting in positive (vs. neutral/negative) sentiment regarding the eyetracking videos with CRR (χ^2^(2)=7.57, *p*=.03).

**Conclusion:**

The ECG training significantly improved participants’ ECG interpretation skills, with a strong start in the training session playing a key role in these improvements. Student feedback indicated that certain subgroups, particularly those with prior cardiological experience, may derive a greater self-reported benefit from the EYE-ECG2 videos.

## Zusammenfassung

**Zielsetzung:**

Ziel dieser Studie war es, die Wirksamkeit einer angepassten Eye-Tracking-Video-Intervention (EYE-EKG2) zur Verbesserung der EKG-Befundungskompetenz von Medizinstudierenden zu untersuchen. Aufbauend auf der Grundlagenstudie EYE-EKG1 [Scherff et al. GMS J Med Educ. 2024], in welcher der Nutzen einer Modellierung von Augenbewegungen durch Experten und Cued Retrospective Reporting (CRR)-Audiokommentaren für den Erwerb von EKG-Befundungskompetenz getestet wurde, werden in dieser Folgestudie Modifikationen eingeführt, die die Lernergebnisse optimieren sollen.

**Methoden:**

Es wurde eine randomisierte kontrollierte Studie mit Medizinstudierenden (*N*=94) durchgeführt, welche entweder der Kontrollgruppe (TAU) mit Standard-EKG-Befundungsstraining, bestehend aus einer validierten Reihe von 4 EKG-Fällen für die EKG-Befundung zugeordnet wurden, oder der Interventionsgruppe (INT), welche zusätzlich das EYE-EKG2-Video erhielt. Das EYE-EKG2-Video optimierte die gezeigten EKG-Blickhinweise und Cued Retrospective Reporting (CRR) eines erfahrenen Kardiologieexperten. Die Studierenden-Performanz wurde Prä- und Post-Training erhoben. Die Daten wurden hinsichtlich einer Verbesserung der Befundungskompetenz, Entscheidungsprozessen und Studierenden-Feedbacks ausgewertet.

**Ergebnisse:**

Die Ergebnisse der vorangegangenen Studie konnten erfolgreich repliziert werden und zeigten einen insgesamt signifikanten Lernvorteil durch die Fälle und eine moderate, jedoch nicht signifikante Tendenz zu INT>TAU (ΔM=0.80-2.42%; *p*=.-79-.30). Die randomisierte Fallpräsentation schwächte die besondere Rolle von Lernfall 1 ab, welche zuvor in EYE-EKG1 beobachtet wurde. Die Untersuchung des studentischen Feedbacks ergab eine überwiegend positive oder neutrale Bewertung (74%), und eine vorherige kardiologische Famulatur war ein Unterscheidungsmerkmal, welches zu einer positiven (vs. neutralen/negativen) Einschätzung der Eyetracking-Videos mit CRR führte (χ^2^(2)=7.57, *p*=.03).

**Schlussfolgerung:**

Das EKG-Training verbesserte die EKG-Befundungskompetenz der Teilnehmenden signifikant, wobei ein gelungener Einstieg in das Training eine Schlüsselrolle für diese Verbesserungen spielte. Das Feedback der Teilnehmenden deutet darauf hin, dass bestimmte Untergruppen, insbesondere diejenigen mit kardiologischer Vorerfahrung, in der Selbstwahrnehmung einen größeren Nutzen aus den EYE-EKG2-Videos ziehen.

## 1. Introduction

Electrocardiography (ECG) interpretation is a critical skill in the medical curriculum, recognized as an Entrustable Professional Activity that students must master before graduation [1], [2]. Yet, with no gold standard for teaching ECG interpretation [3], performance results are frequently poor [4], leaving significant scope for developing and improving new teaching tools. 

The original EYE-ECG study [5] explored an innovative method using eye-tracking videos combined with Cued Retrospective Reporting (CRR; as in e.g., [6], [7], [8], [9], [10], [11]) to enhance ECG interpretation skills. The theoretical underpinning of this approach is to employ Eye Movement Modelling Examples (EMMEs) in order to enhance ECG interpretation training, leveraging eye-tracking technology to replicate expert visual search patterns, aiding novices in efficient diagnostic learning [12], [13], [14]. Previously successful in histology and radiology, EMMEs have also improved clinical reasoning and adaptive expertise in medical imaging [15], [16], [17]. AI integration in automated ECG analysis benefits from expert gaze modelling, yet challenges remain, including variability in eye movements and accessibility of tracking technology [18], [19], [20]. While EMMEs enhance medical education, limitations include differences in expert gaze patterns, cognitive overload, and transferability of skills, requiring further study [21], [22], [23]. In the following, we employ the term “eye-tracking videos” as a practical application of EMMEs.

CRR asks from an expert to review their eye-tracking patterns during ECG interpretation and verbally explain their thought process. CRR, developed as a retrospective think-aloud technique, allows experts to articulate their reasoning without disrupting the original expert task. Together, eye-tracking and CRR could offer students both visual and verbal insights, potentially enhancing their understanding of effective expert ECG interpretation strategies.

The predecessor study provided tentative evidence suggesting that the combination could be harnessed to positively influence ECG interpretation skills among medical students. However, the results also highlighted areas for improvement, leading to the development of the current EYE-ECG2 study. Three key previous observations informed the design of this follow-up research.

Although the eye-tracking videos with CRR were promising, it became apparent that modifying these videos could potentially enhance their effectiveness. This follow-up study aimed to explore whether adjustments in the presentation of these videos, synchronising the eye-tracking focus spot with their verbal explications from cued retrospective reporting, could lead to better outcomes.

One of the most intriguing findings from EYE-ECG1 was the particular relevance of the first clinical training case, which involved myocardial infarction. This observation gave rise to the hypothesis that posterior myocardial infarction might serve as *gatekeeper content* – a critical component of ECG interpretation that must be understood before students can progress to more complex cases. Alternatively, it was hypothesized that success in the first case might have a motivational impact, setting the tone for subsequent learning. EYE-ECG2 seeks to formally test these alternative explanations.

Finally, the original study identified a divergence in informal student feedback regarding the use of the EYE-ECG video. Some students found them extremely helpful, while others did not. EYE-ECG2 aims to systematically investigate the factors contributing to these differing perceptions, examining whether specific student characteristics might explain why certain students respond more positively to this teaching method than others.

Building on the findings of the predecessor study, EYE-ECG2 aims to refine the use of eye-tracking videos with CRR and explore their impact on ECG interpretation skills among medical students. The study will focus on three main objectives:

1. assessing the impact of modified eye-tracking videos on student learning outcomes,
2. investigating the specific role of the first clinical case in ECG interpretation skills acquisition, and
3. analysing student feedback to identify factors that influence their reception of this novel teaching method.

## 2. Materials and methods

### 2.1. Design

**Figure 1:**
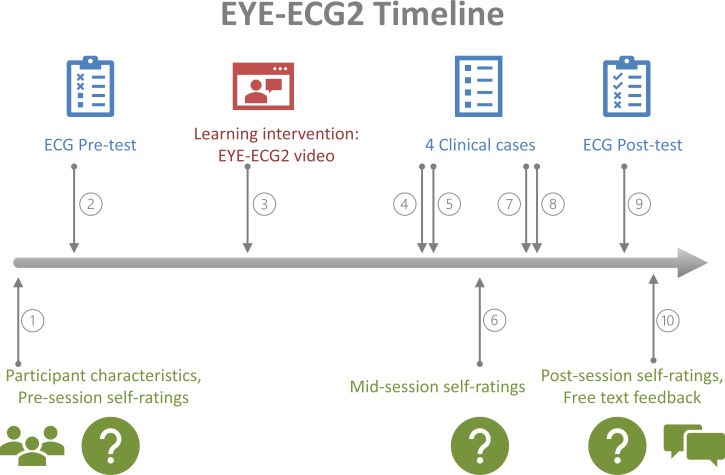
Overview of all EYE-ECG2 components and order of presentation

The study (see figure 1 (Fig. 1)), henceforth referred to as *EYE-ECG2*, was designed as a follow-up study to the EYE-ECG1 study [5]. Identically for both studies, in a randomised controlled trial of a learning study, effects of expert eye-tracking videos with *cued retrospective reporting (CRR)* on medical students’ ECG interpretation skills were the focus of investigation. These recordings served as the learning intervention *(EYE-ECG video)* in the* intervention group (INT)*, while the *training as usual group (TAU)* received the same ECG training session without seeing the videos. Participants completed the ECG training session online under standardised, distraction-free conditions. All training components were presented in a fixed sequence, and once an answer was submitted, it could not be changed. At the beginning of the session (component 1, see figure 1 (Fig. 1)), participants provided demographic information and completed self-ratings regarding their interest in ECGs and confidence in their learning strategies. They also rated their current motivation and restedness. Next (component 2), participants completed an ECG pre-test assessing theoretical ECG knowledge. The learning intervention (component 3) involved viewing the updated EYE-ECG2 video (modification 1), which provided a revised instructional approach to systematic ECG interpretation. Participants were required to watch the video at least once but could revisit it as needed. Following the video, participants engaged with four clinical case vignettes (components 4, 5, 7, and 8). Each case presented an ECG scenario accompanied by several test questions targeting different aspects of ECG interpretation, including heart rate, rhythm, axis, intervals, and amplitudes. In contrast to the previous study, the presentation order of the four clinical cases was randomised across participants to minimise sequence effects (modification 2). After completing the first two clinical cases, participants filled out mid-session self-ratings (component 6) concerning the accessibility of the material and their flow experience during learning. Motivation and restedness were also re-assessed. After all four clinical cases, participants proceeded to the ECG post-test (component 9), which involved applied diagnostic tasks based on practical ECG examples. Finally (component 10), participants completed post-session self-ratings evaluating their perceived learning gain and provided free-text feedback. A final rating of motivation and restedness was also collected.

The present EYE-ECG2 study built on two specific recommendations identified in EYE-ECG1, to modify the videos using cued retrospective reporting and to randomise learning cases within the learning intervention. EYE-ECG1 will be reintroduced here briefly for the benefit of the reader.

### 2.2. Measures

#### 2.2.1. The EYE-ECG2 video

**Figure 2:**
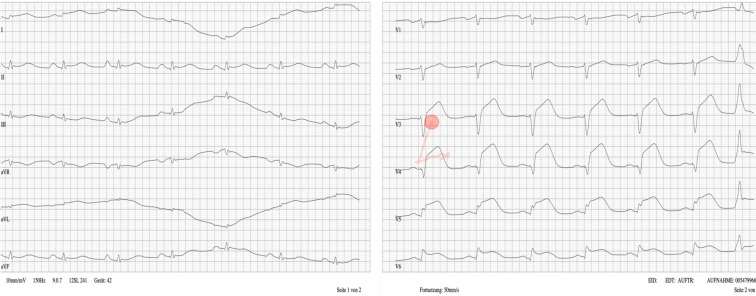
Screenshot of one of the ECGs used in the EYE-ECG2 video. Compared to the previous study, Modification 1 pauses the video at this moment. Audio commentary during this video frame: “Here the focus is primarily on the ST elevations in the anterior wall leads from V3 to V6...[video moves on -- not shown --] and then quickly in comparison, what is in the limb leads...”

Previously, for EYE-ECG1, a video was developed that later on served as the novel learning intervention for the INT group (please refer to [5] for full details). Initially, 15 authentic patient ECGs representing a range of cardiological diagnoses were selected. These were shown to a senior cardiology expert, who silently and freely interpreted all ECGs while wearing a head-mounted eye-tracker. Next, the recorded eye movements were projected onto the respective 15 ECG images, visualised as a moving red focus spot with a trail marking previously viewed locations (see figure 2 (Fig. 2)). Marked spots dynamically varied in size corresponding to longer or shorter inspection during the expert viewing session. ECGs with superimposed eye movements were then once again shown to the same expert, this time recording audio commentary using the silent videos as cues for verbal explications of their own prior viewing behaviour and diagnostic processes (=cued retrospective reporting, CRR; adapted from [24]). Finally, visual and auditory expert diagnostic interpretation on all ECGs was combined into a single video (EYE-ECG1 and -2 video for study 1 and 2, respectively).

#### 2.2.2. EYE-ECG2 modification 1

Student feedback from the first study indicated a demand of students to “pause” the eye gaze during crucial moments of the expert’s diagnostic process. To clarify, this did not refer to the ability to pause the video entirely (which was already both possible and permitted during the first study). Rather, multiple participants suggested holding the focus spot in place while corresponding verbal explanations would be continuing. The first modification within EYE-ECG2, compared to its predecessor, was thus to alter the EYE-ECG video, synchronising the expert eye gaze with the relevant content of the audio track. This adjustment was performed on pre-existing materials, i.e., no new expert eye-tracking or audio recordings were collected. Synchronisation was achieved by retrospectively identifying moments where visual focus and verbal commentary closely corresponded, and by holding the focus marker stationary during the associated verbal explanation. Specifically, visual triggers for commentary – such as the visual marker focusing on the QRS complex in lead II – were matched with temporally proximal verbal chunks of information (e.g., “normal electrical axis” a few milliseconds later), so that the gaze pattern remained visually stable while the verbal explanation fully addressed the diagnostic feature.

The close temporal association between eye movements and verbal comments in the original material facilitated this synchronisation. Depending on the complexity of the ECG findings, the duration of the verbal commentary either approximately matched or exceeded the time originally needed for silent visual scanning. Separate recording of eye-tracking and commentary ensured that natural gaze behavior was preserved and was not artificially prolonged or altered by the process of verbalisation. During the synchronised segments, the video was “paused” (i.e., the marker held in place) for the duration of the verbal explanation, and then continued normally.

Importantly, this synchronisation posed the only modification undertaken: all visual and auditory material was preserved (i.e., none was cut or removed), and the order of ECGs, viewing patterns, and verbal comments remained identical to that of the previous study. No parts of the expert commentary or eye-tracking data were shortened, extended, or artificially rearranged.

This approach mirrored principles used in educational videos, where dynamic visualisations are temporarily stabilised to allow learners to process complex information. Participants were also able to freely pause, rewind, or re-watch the EYE-ECG2 video at their discretion, for example to take notes, similar to how students interact with online instructional videos. Nevertheless, it was mandatory for all participants to view the entire video at least once without interruption; adherence to this instruction was verified by tracking the dwell time on the study platform.

#### 2.2.3. The ECG interpretation training session

**Figure 3:**
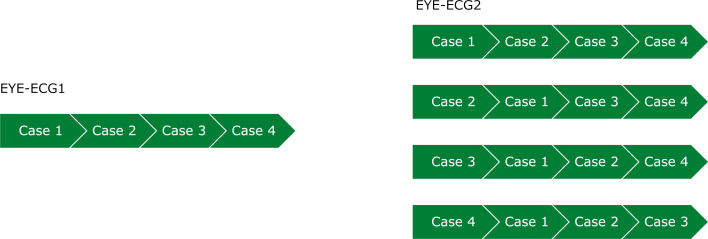
Modification 2. Illustration of the order of presentation of clinical cases 1-4 within the training session

The ECG training session using four clinical cases that was given to all participants of the EYE-ECG2 study was identical to that of the first study [5], save for one modification (see figure 3 (Fig. 3)). To briefly revisit, the student ECG training had been developed and utilised over the course of several previous studies [25], [26], [27] and consists of 9 (TAU) or 10 (INT, including the EYE-ECG2 video) components, as shown above in figure 1 (Fig. 1).

#### 2.2.4. EYE-ECG2 modification 2

Statistical analysis of the EYE-ECG1 data using multiple regression models suggested a somewhat distinctive role of clinical case 1 in predicting post-session ECG interpretation skills of participating students. However, within the first study, the source of this finding could not be pinpointed. The second modification within EYE-ECG2, therefore, was to randomise presentation of this clinical case and to observe whether the previously detected effect would subside. This was operationalised as presenting participants with either case 1, 2, 3, or 4 as their first case, followed by the remainder of the cases in original order (e.g., case 3, ---1, 2, 4). Allocation to the four possible orders of presentation was quasi-random, i.e., maintaining an even number of participants per order, and across training (INT vs. TAU) groups.

*Participant characteristics* collected were sex (male/female), age (years), years in education (no.), prior medical vocational training (yes/no), subject-related semester (no.), prior ECG experience (no.), prior cardiological clerkship (yes/no).

*Self-rated scales* administered were confidence in personal learning strategy (0-100%), interest in ECGs (0-100%), current motivation pre-/mid-/post- session (0-100%), current restedness pre-/mid-/post- session (0-100%), accessibility of the material mid-session (0-100%), flow state mid-session (0-100%), and self-rated benefit from the training post-session (0-100%).

*ECG skill measures* were composed of a pre-test measuring theoretical ECG knowledge, 4 *clinical cases *centring on the visual and clinical interpretation of a patient’s ECG, and a post-test presenting quick practical ECG scenarios based on 9 authentic patient ECGs. 

As discussed at length elsewhere [5], there is no universal guideline how to best and meaningfully measure students’ ECG skill gain. To this end, EYE-ECG1 previously developed 3 scoring variants capturing different aspects of ECG interpretation skills for further exploration, that were again utilised in EYE-ECG2. The basic score (BS) awarded points for each correctly identified ECG feature (0-100%); the relative score (RS) evaluated correctly against falsely chosen options (-100-100%); the conservative score (CS) counted only fully correctly answered questions (0-100%). As was argued, these scores might aid the investigation of a student’s learning journey from feature detection, via self-monitoring, to clinical competence.

#### 2.2.5. Free text feedback

At the very end of the training session, participants saw a simple prompt (“Please give feedback on…”) to provide feedback

1. on the EYE-ECG2 video (INT only) and
2. on the ECG training session.

To also allow some statistical contemplation of the comments, a quick manual categorisation of the tone of individual comments into negative, neutral, or positive was undertaken, creating the variable sentiment (-1; 0; 1; respectively).

#### 2.2.6. Procedure

Identical to the predecessor study, EYE-ECG2 required of its participants a prior successful completion of their university cardiology module and that students were not yet fully qualified medical doctors. All participants were randomly assigned to either receive the EYE-ECG2 video training or training as usual (*N*=94; INT *n*=47; TAU *n*=47) within the online training session. As before, the intervention group was instructed to fully watch the 12m 04s EYE-ECG2 video at least once and training components were worked through in fixed order in this ~2-hour training session.

## 3. Results

### 3.1. Sample characteristics, self-ratings and manipulation checks

The sample was composed of 59% females and 41% males aged *M*=23.83 (*SD*=3.67) years, and *M*=18.53 (*SD*=2.99) years in education. In line with recruiting only those having passed their cardiology exam, current semester was *M*=9.43 (*SD*=1.66) equating to 4^th^/5^th^ year of studies. A prior medical vocational training had been completed by 21% and a prior cardiological clerkship was done by 21%. Interestingly, with the study being conducted closely after COVID-19 related restrictions at university had been lifted, equal proportions of students reported never having received *any* dedicated ECG training, having received ECG training during their studies and through some combination of university, online, and external sources (33%/33%/34%). Self-estimated mean number of previously independently interpreted ECGs was *M*=74.80, with large individual differences (*SD*=516.47 driven by very few students with high self-reports; and thus lower Mdn=10.00). Self-rated interest in ECG interpretation was high (*M*=75.00%, *SD*=13.89%), students were confident in their personal learning strategy (*M*=67.18%, *SD*=10.87%), the learning content of the study was judged overall as accessible (*M*=47.82%, *SD*=14.66%), flow state indicated moderate mental effort was required (*M*=53.85%, *SD*=16.41%), and post-training benefit was substantial (*M*=46.93%, *SD*=14.24%). 

As expected, restedness (pre=58.57%±23.49%, mid=44.67%±23.19%, post=37.92%±23.92%) and motivation (pre=74.70%±19.83%, mid=60.12%±22.21%, post=47.08%±26.15%) scores reduced significantly over the course of the session (all paired t-tests significant; motivation: pre-mid* t*(93)=-6.50, *p*<.04*10^-8^, pre-post *t*(91)=-9.95, *p*<.03*10^-13^, mid-post *t*(91)=-8.06, *p*<.03*10^-10^; restedness: pre-mid *t*(93)=-7.56, *p*<.03*10^-9^, pre-post* t*(91)=-8.16, *p*<.02*10^-10^, mid-post *t*(91)=-3.48, *p*<.001). 

Working time was *M*=103.46min (*SD*=22.60) for the entire training session. Time spent on the 12m 04s EYE-ECG2 video by the intervention group was *M*=14.36min (*SD*=3.06). All manipulation checks comparing INT against TAU (i.e., session duration, working speed*post-test performance, random group allocation all nonsignificant in t-tests; cf. EYE-ECG1) were satisfactory. In comparison, the sample closely resembled that of the EYE-ECG1 study.

### 3.2. Modification 1: Effects of the EYE-ECG2 video on ECG interpretation skills

**Figure 4:**
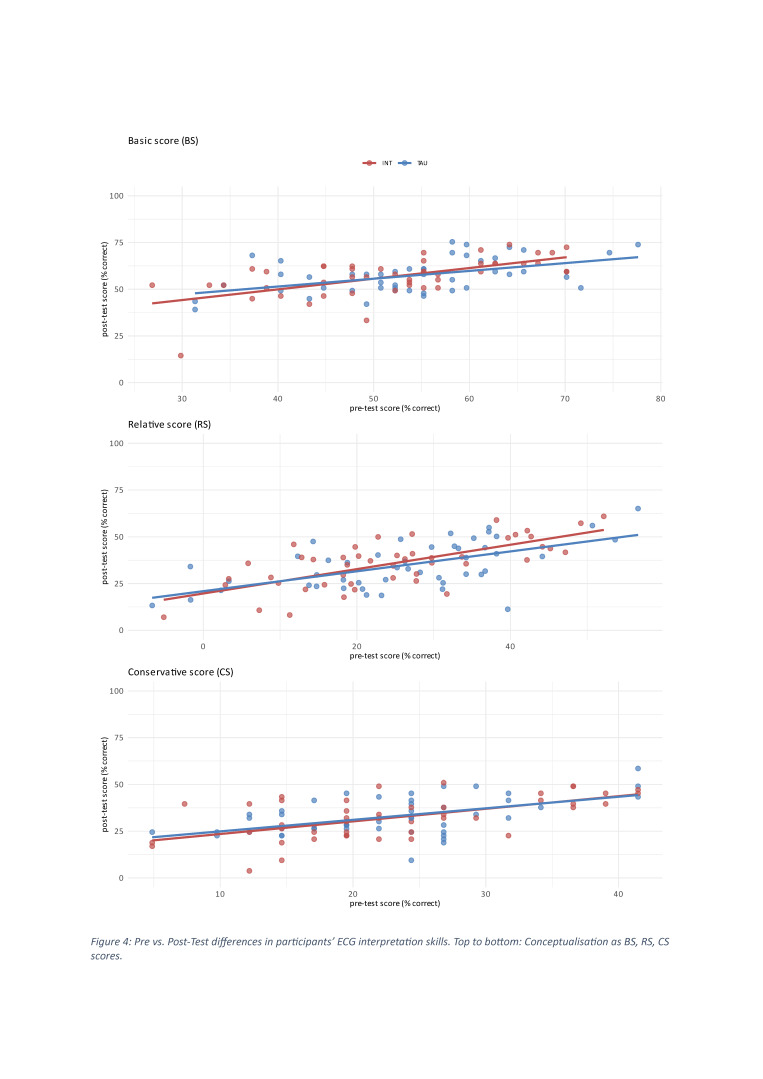
Pre vs. Post-Test differences in participants’ ECG interpretation skills. Top to bottom: Conceptualisation as BS, RS, CS scores

Paired t-tests show the training session significantly and moderately improved participant ECG interpretation skills (ascending lines in figure 4 (Fig. 4); pre-post gain BS=3.98%±9.82%, *t*(93)=3.93, *p*<.02*10^-2^; RS=9.89%±11.20%, *t*(93)=8.56, *p*<.03*10^-11^; CS=9.70%±9.20%, *t*(93)=10.22, *p*<.03*10^-14^). Regarding the effects of expert eye-tracking videos with cued retrospective reporting on student ECG interpretation skills (i.e. the effect of the first modification of the study, namely the synchronised EYE-ECG2 video specifically), the three pre-post difference scores were BS ΔM=1.60%±2.03%, RS ΔM=2.42%±2.31% and CS ΔM=0.80%±1.91%, conferring a nonsignificant tendency towards better performance using the video (red vs. blue lines in figure 4 (Fig. 4); BS Welch-*t*(91.67)=0.79, *p*=.43; BS Welch-*t*(90.07)=1.05, *p*=.30; CS Welch-*t*(91.70)=1.15, *p*=.67).

### 3.3. Modification 2: Model-fitting of best predictors for basic, relative, and clinical ECG interpretation skill scores

Model-fitting was replicated using the identical analytical strategies from EYE-ECG1 (as described in [5], i.e., multiple regression using stepwise backward model selection based on Akaike information criterion (AIC)). Full results are shown in attachment 1 point A for completeness and in order to permit direct comparison to the preceding study. However, for brevity and clarity, portrayal here will be limited to that of the role of clinical case 1, which represents the second modification of the study: In this respect, randomising the order of presentation of clinical case 1 resulted in an attenuated effect and even in the removal of case 1 from the best-fitting model for both the BS and RS model (for predictors included in the final models refer to table 1 (Tab. 1)).

### 3.4. Insights from free text student feedback

Overall, 35 of 47 students (74%) of the INT group followed the request for written feedback on the EYE-ECG2 video. Of those who did provide feedback, sentiment was mostly positive (45% positive, 29% neutral, 26% negative). Next, participant characteristics were evaluated against sentiments using chi-square tests (nominal variables) and spearman correlations (numerical variables). Results show that neither sex, age, years in education, prior medical vocational training, subject-related semester, or prior ECG experience were significantly associated with sentiment. Singularly prior cardiological clerkship was a distinguishing factor in how the EYE-ECG2 video was received by participants (χ^2^(2)=7.57, *p*=.03). Notably, all of the negative sentiments were given by students without the experience of a prior cardiological clerkship. Hereafter, extracts of actual student feedback are shown.

Participants giving positive feedback described liking that “the red spot made it easy to follow what to look for at that moment”, “the eye movements were good to follow along”, “seeing the focus of the expert was helpful”, “it was good overall that so many different pathologies were discussed”, “the video was instructive overall as many different cases were shown”, “I liked the explanations”, “the video really helped”, “great use of the eye movements and right to the point”, “I especially liked the video with the recorded eye movements of the expert”, “structured approach, focus on important features in the ECG”, “that you could see the eye movements of the lecturer”.

Neutral feedback stated that “In principle, I found the eye movements interesting. However, they led to me not being able to look at the ECG at my own pace, but instead having to constantly follow the point that was moving too quickly for my skill level”, “eye movements partly without comment; sudden end of the presentation of the case”, “eye movements should be more in sync with the spoken text. Also, the eye movements are not always indicative of the directive analysis. It is primarily explained what is being looked at (descriptive) and not why (explanatory).”

Negative feedback noted that “explain what the dot with the pink string behind it is: eye movements of the physician?”, “extremely high amount of material in very little time and all in one go. I think it's not bad for answering questions within the next hour, but when you repeat the same test a week later, none of it is there anymore”, “The eye movements are useless. A simple pointer would be more helpful”.

## 4. Discussion

The primary objective of the EYE-ECG2 study was to explore whether enhancements to eye-tracking videos with Cued Retrospective Reporting (CRR) could improve ECG interpretation skills among medical students. Building on the findings of the predecessor study, EYE-ECG1, which introduced the novel use of eye-tracking technology in ECG training, this study aimed to refine the learning intervention by synchronising eye movements with expert commentary and randomising case presentation order. The study also sought to analyse the feedback from participants to better understand the factors that influenced their reception of the EYE-ECG videos.

The findings from EYE-ECG2 largely replicated the results of the original study, tentatively confirming that the use of eye-tracking videos with CRR can positively impact students' ECG interpretation skills. However, the enhancements made in this follow-up study, particularly the synchronisation of eye movements with the corresponding expert commentary, did not lead to a greater improvement in learning outcomes compared to the original EYE-ECG1 video. The slight, nonsignificant trend of better performance in the group receiving the EYE-ECG video suggests that while the video format was beneficial, the specific modifications may not have provided additional value.

This aligns with broader research on Eye Movement Modeling Examples (EMMEs), which suggests that their effectiveness depends on cognitive load, processing depth, and the timescale of their impact [14], [20], [28]. While EMMEs have been shown to aid attentional guidance and gaze alignment, their immediate effects on diagnostic accuracy remain inconsistent, particularly when learners engage in surface-level processing [12], [29]. In this study, the absence of a direct performance gain could be attributed to cognitive overload, as novices may struggle to integrate expert gaze patterns efficiently without additional scaffolding [30], [31]. This reinforces the idea that EMMEs may primarily support long-term adaptation rather than immediate skill transfer, with improvements in visual search behavior preceding measurable diagnostic gains [32], [33]. Building on this, prior research suggests that multi-modal approaches, such as combining EMMEs with Cued Retrospective Reporting (CRR), can help mitigate cognitive load by engaging both visual and verbal cognitive channels [13], [16]. Studies in medical training and multimedia learning have shown that verbal-visual integration enhances cognitive processing, reduces extraneous load, and improves knowledge retention [28], [33], [34]. Specifically, pairing expert gaze guidance with verbal justifications has been found to enhance diagnostic accuracy and problem-solving efficiency [12], [14], [35]. Additionally, structuring complex visual input into segmented, expert-guided explanations can help optimise working memory resources, fostering long-term skill acquisition [36], [37]. Future research should further examine how these elements interact to refine EMME-based ECG training, balancing complexity, cognitive demands, and sustainable learning outcomes, while also considering the role of learner engagement and interest. The latter, as replicated in this study and discussed below, could be a key driver of perceived benefit and positive evaluations.

Another key insight from this study was the impact of randomising the presentation of the clinical learning cases. Previously the first clinical case was found to have a noticeably strong effect on post-test performance, suggesting that it might act as a gatekeeper for subsequent learning. In EYE-ECG2, after randomising the order in which cases were presented, this effect was attenuated. This finding supports the hypothesis that the initial success in the (i.e. any) first-presented case, rather than the specific content of the myocardial infarction case, was crucial in setting the tone for the students’ subsequent performance. Thus, the positioning of learning materials in an ECG training session was evident, emphasising the need for careful selection and placement of critical content early in the training process.

Given the consistent finding across EYE-ECG1 and EYE-ECG2 that interest in ECGs predicts performance, it is worth exploring why this is the case and what aspects of ECG interpretation students find particularly engaging. In this context, the way ECG content is presented – especially through eye-tracking and CRR – may play a role in shaping engagement, though its impact likely depends on other factors, as reflected in students’ varied perceptions of the EYE-ECG2 videos. The sentiment analysis offers a quick preliminary overview of how students responded, informing on general trends in their feedback. Although the majority of students rated the EYE-ECG2 videos positively, the study flagged that relevant prior experience, specifically a cardiological clerkship, significantly influenced students’ perceptions. Students who had completed a cardiological clerkship were more likely to appreciate and subjectively benefit from the eye-tracking videos with CRR. This suggests that a certain level of real-world exposure to cardiological expertise may be necessary for students to fully engage with and benefit from the detailed visual and verbal information provided in the EYE-ECG videos. On the other hand, students without such prior experience might find the videos less accessible or beneficial, potentially due to the complexity of the content and the advanced level of expert reasoning presented.

### 4.1. Limitations

While the EYE-ECG2 study replicated some of the benefits observed in the original EYE-ECG intervention, some limitations must also be considered. First, the study’s sample size may have been insufficient to detect small but meaningful differences between the intervention and control groups, which could explain the nonsignificant trends observed. Second, the study focused and explored self-reported feedback and its sentiment, and at this point did not link it to actual differences in performance. This aspect could be further explored in the future. Additionally, incorporating richer qualitative methods such as focus groups, semi-structured interviews, usability testing, or think-aloud protocols, could provide deeper insights into how students engage with the intervention. In particular, understanding how learners with different levels of ECG expertise interact with novel teaching methods may help to develop learner profiles that capture individual learning pathways, ultimately informing how such interventions can be best tailored to diverse student needs.

## 5. Conclusion

In conclusion, while the EYE-ECG2 study successfully replicated the benefits of the original EYE-ECG intervention, the modifications made did not result in significant additional gains. The findings underscore the importance of case order in ECG training and suggest that ECG tutors should carefully consider the sequencing of learning materials to optimise student outcomes. Additionally, the differential impact of the EYE-ECG videos based on students' prior clinical experience highlights the need for future research to explore how different levels of ECG experience influence the reception and effectiveness of eye-tracking with CRR as a novel teaching method. Specifically, it would be of interest to investigate further how students with low, moderate, and high levels of ECG expertise usually engage with different types of learning materials and how this affects their expectations of how EYE-ECG should be tailored to best meet the needs of such diverse learner profiles. 

## Authors’ ORCIDs

- Aline D. Scherff: [0000-0002-7420-2292]
- Stefan Kääb: [0000-0001-8824-3581]
- Martin R. Fischer: [0000-0002-5299-5025]
- Markus Berndt: [0000-0002-4467-5355]

## Competing interests

The authors declare that they have no competing interests. 

## Supplementary Material

Comprehensive study results

## Figures and Tables

**Table 1:**
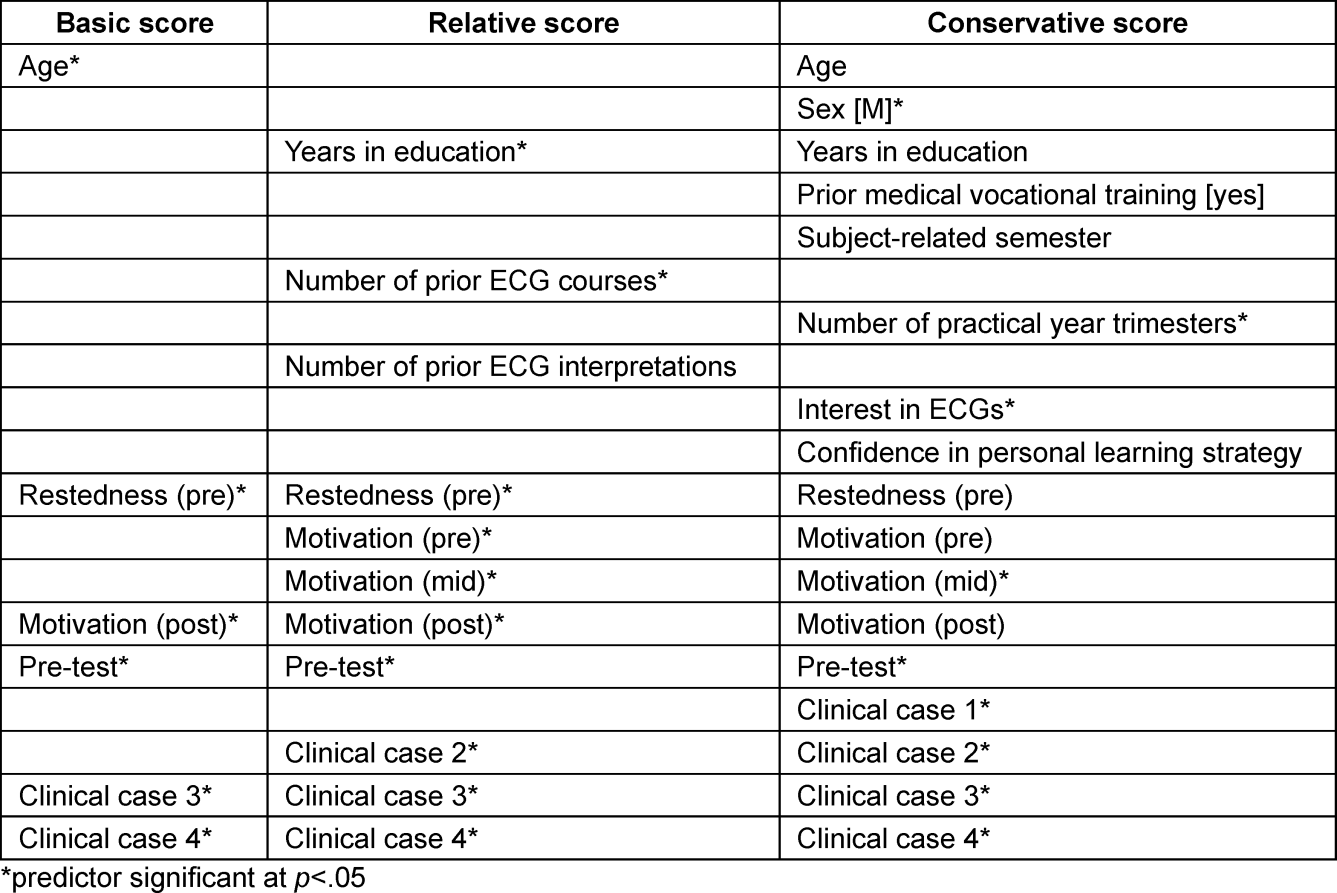
List of predictors included in the three final regression models
