# Supplementary material for: EYE-ECG2: Addressing medical student feedback in an RCT with eye-tracking videos featuring cued retrospective reporting and modified learning sequences for ECG interpretation skills training
Source: GMS J Med Educ. 2025 Sep 15;42(4):Doc47. doi: 10.3205/zma001771 (PMC12527390; doi:10.3205/zma001771)
Supplement: Comprehensive study results [file JME-42-47-s-001.pdf]

Attachment 1: Comprehensive study results

	Specific ECG interpretation skill →	I. Basic score				II. Relative score				III. Conservative score			
		full model		final model		full model		final model		full model		final model	
conceptual grouping	↓ **Included variables (predictors)**	**beta**	**p-value**	**beta**	**p-value**	**beta**	**p-value**	**beta**	**p-value**	**beta**	**p-value**	**beta**	**p-value**
	(intercept)	-3.35	.79	**12.79**	**< .05**	-28.98	.06	-13.79	.06	**26.24**	**.04**	**24.36**	**.01**
Learning intervention	group *[INT]*	0.56	.76			-0.02	.99			-1.15	.53		
participant characteristics/obj. characteristics	age	0.47	.30	**0.48**	**.01**	0.07	.90			-0.70	.13	-0.55	.19
	sex *[M]*	1.18	.54			1.22	.60			3.06	.10	**3.69**	**.02**
	years in education	-0.27	.61			0.43	.51	**0.76**	**.01**	0.98	.07	0.83	.08
	prior medical vocational training*[yes]*	-0.01	.99			1.92	.53			4.82	.06	3.03	.17
	subject-related semester	-0.19	.76			-0.29	.70			-0.76	.24	-0.86	.13
	number of prior ECG courses	-2.26	.10			**-4.59**	**.007**	**-3.90**	**.008**	-1.66	.22		
	number of practical year trimesters	1.22	.63			3.85	.22			**6.13**	**.01**	**6.47**	**.004**
	cardiological clerkship *[yes]*	-1.62	.47			-1.90	.49			-1.39	.53		
	number of prior ECG interpretations	< 0.01	.40			0.00	.25	**< 0.01**	**.10**	0.00	.75		
subj. learning characteristics	interest in ECGs	0.05	.56			0.04	.67			0.11	.13	**0.13**	**< .05**
	confidence in personal learning strategy	0.10	.30			0.12	.30			-0.12	.21	-0.13	.10
	accessibility of material	0.12	.30			0.14	.30			0.14	.22		
	flow state	0.08	.29			0.08	.33			-0.04	.55		
	self-rated benefit of training session	-0.01	.91			0.00	.97			-0.04	.73		
participant involvement	restedness (pre)	**-0.12**	**< .05**	**-0.08**	**.03**	**-0.16**	**.03**	**-0.11**	**.01**	-0.08	.17	-0.05	.17
	restedness (mid)	0.12	.11			0.13	.14			0.05	.54		
	restedness (post)	-0.07	.24			-0.03	.65			-0.01	.90		
	motivation (pre)	0.06	.26			0.12	.07	**0.12**	**.02**	0.07	.18	0.07	.11
	motivation (mid)	-0.08	.30			-0.17	.08	**-0.17**	**.03**	**-0.16**	**.04**	**-0.15**	**.03**
	motivation (post)	0.10	.13	**0.06**	**.04**	0.10	.24	**0.14**	**.03**	0.04	.50	0.07	.22
learning content	pre-test	**0.20**	**.04**	**0.25**	**.002**	**0.24**	**.02**	**0.28**	**.002**	**0.46**	**.0002**	**0.49**	**.009*10^{-3}**
	clinical case 1	0.04	.52			-0.01	.89			**0.97**	**.004**	**0.95**	**.002**
	clinical case 2	0.11	.21			0.17	.06	**0.15**	**.05**	**0.22**	**.02**	**0.23**	**.01**
	clinical case 3	0.15	.11	**0.20**	**.004**	**0.25**	**.02**	**0.30**	**.001**	**0.23**	**.04**	**0.22**	**.03**
	clinical case 4	**0.18**	**.04**	**0.16**	**.03**	**0.24**	**.03**	**0.20**	**.02**	**0.28**	**.005**	**0.24**	**.008**
	Summary statistics:	R^2 = .56, R^2_{adj} = .37, F(26,63) = 3.05, p < .02*10^{-2}, R^2 = .47, R^2_{adj} = .43, F(12,83) = 12.09, p < .01*10^{-7}				R^2 = .66, R^2_{adj} = .52, F(26,63) = 4.73, p < .02*10^{-5}, R^2 =.62, R^2_{adj} = .57, F(11,78) = 11.78, p < .01*10^{-10}				R^2 = .65, R^2_{adj} = .50, F(26,63) = 4.42, p < .007*10^{-4}, R^2 = .62, R^2_{adj} = .53, F(17,72) = 6.91, p < .02*10^{-7}			

Note: [ ] denotes reference group; calculations of scores (cf. statistical analyses section) for pre-test and clinical cases 1-4 corresponded model-wise to the specific (BS, RS, CS) outcomes regressed upon; model selection stepwise backwards *via Akaike Information Criterion (AIC); significant predictors are bold*

Attachment 1 to Scherff AD, Kääb S, Fischer MR, Berndt M. *EYE-ECG2: Addressing medical student feedback in an RCT with eye-tracking videos featuring cued retrospective reporting and modified learning sequences for ECG skills training*. GMS J Med Educ. 2025;24(4):Doc47. DOI: 10.3205/zma001771
